# Shifts in Soil Structure, Biological, and Functional Diversity Under Long-Term Carbon Deprivation

**DOI:** 10.3389/fmicb.2021.735022

**Published:** 2021-09-14

**Authors:** Paul B. L. George, David B. Fidler, Joy D. Van Nostrand, Jonathan A. Atkinson, Sacha J. Mooney, Simon Creer, Robert I. Griffiths, James E. McDonald, David A. Robinson, Davey L. Jones

**Affiliations:** ^1^School of Natural Sciences, Bangor University, Bangor, United Kingdom; ^2^UK Centre for Ecology & Hydrology, Bangor, United Kingdom; ^3^Département de Médecine Moléculaire, Université Laval, Quebec City, QC, Canada; ^4^Institute for Environmental Genomics, The University of Oklahoma, Norman, OK, United States; ^5^School of Biosciences, University of Nottingham, Sutton Bonington Campus, Sutton Bonington, United Kingdom; ^6^SoilsWest, UWA School of Agriculture and Environment, The University of Western Australia, Perth, WA, Australia

**Keywords:** agricultural grassland, anaerobic respiration, bare fallow, carbon residence time, soil microbial community

## Abstract

Soil organic matter is composed of a variety of carbon (C) forms. However, not all forms are equally accessible to soil microorganisms. Deprivation of C inputs will cause changes in the physical and microbial community structures of soils; yet the trajectories of such changes are not clear. We assessed microbial communities using phospholipid fatty acid profiling, metabarcoding, CO_2_ emissions, and functional gene microarrays in a decade-long C deprivation field experiment. We also assessed changes in a range of soil physicochemical properties, including using X-ray Computed Tomography imaging to assess differences in soil structure. Two sets of soils were deprived of C inputs by removing plant inputs for 10 years and 1 year, respectively. We found a reduction in diversity measures, after 10 years of C deprivation, which was unexpected based on previous research. Fungi appeared to be most impacted, likely due to competition for scarce resources after exhausting the available plant material. This suggestion was supported by evidence of bioindicator taxa in non-vegetated soils that may directly compete with or consume fungi. There was also a reduction in copies of most functional genes after 10 years of C deprivation, though gene copies increased for phytase and some genes involved in decomposing recalcitrant C and methanogenesis. Additionally, soils under C deprivation displayed expected reductions in pH, organic C, nitrogen, and biomass as well as reduced mean pore size, especially in larger pores. However, pore connectivity increased after 10 years of C deprivation contrary to expectations. Our results highlight concurrent collapse of soil structure and biodiversity following long-term C deprivation. Overall, this study shows the negative trajectory of continuous C deprivation and loss of organic matter on a wide range of soil quality indicators and microorganisms.

## Introduction

Soil carbon (C) loss is a serious concern for global agricultural management in addition to contributing to climate change (Lal, 2004). It is estimated that soils will lose 55 Pg C globally by 2050, though this will vary across regions (Crowther et al., 2016). Current estimates of soil C stocks in the United Kingdom total 6.72 Gt (Minasny et al., 2017). Bellamy et al. (2005) reported that organic C is lost from soils in England and Wales at a rate of 0.6% per year; UKCEH-Countryside Survey support the loss in agricultural soils (Reynolds et al., 2013), but is yet to find evidence in other habitats. These losses have been ascribed to shifts in land management regimes including increased rates of erosion, livestock stocking, and grassland conversion, although the precise drivers remain unknown.

Soil C comprises a large range of organic compounds with varying levels of accessibility and degradability by soil organisms. Historically a continuum of C forms was defined on these criteria as labile or recalcitrant (Rovira and Vallejo, 2002) based on residence time in soil (Cheng et al., 2007). However, recent work suggests that long-held views of recalcitrance are being superseded by new insights into molecular structure. For example, Lehmann et al. (2020) posit that it is the molecular complexity of soil organic matter, which limits decomposition rates. A wide range of organisms can readily consume common plant-derived compounds like protein, lipids, and cellulose (Romero-Olivares et al., 2017; Ogden et al., 2018). In contrast, more recalcitrant C forms, such as lignin and high molecular weight, complex humic substances reside much longer in soils (Rovira and Vallejo, 2002; Romero-Olivares et al., 2017; Lehmann et al., 2020), and are thought to be decomposed primarily by fungi (Adl, 2003), though recent evidence suggests that saprotrophic fungi are more prominent consumers of labile C than previously understood (de Vries and Caruso, 2016). Thus, as labile and exposed soil C sources are depleted, it is expected that organisms capable of exploiting recalcitrant C forms are more likely to persist.

Anthropogenic processes involving the removal of aboveground plant cover (e.g., bare fallow periods, plowing, sealing, and drought) reduce C inputs and cause net losses of soil organic matter. This has negative consequences on other parts of the soil system. For example, soils under long-term bare fallow typically become progressively more acidic (Paradelo et al., 2013) and lose both aggregate stability (Paradelo et al., 2016) and labile C (Coleman et al., 1997; Barré et al., 2010). In addition, reduced C inputs can lower soil porosity at the μm scale (Bacq-Labreuil et al., 2018). A reduction in pore size and connectivity means that soil microorganisms are faced with less spatial diversity and habitable space, since pores are sites of water and gas exchange (Ruamps et al., 2011). The changes in soil structure under long-term C deprivation are therefore expected to strongly affect the size, composition and activity of soil bacterial and fungal communities (Hirsch et al., 2009, 2017), which subsequently impacts downstream taxa within soil food webs, such as protists.

However, there have been few attempts to reconcile all these disparate aspects of soil degradation through C deprivation under experimental conditions. Protists, which include the largest fraction of primary consumers of the soil biological community (Adl, 2003), have not featured prominently in such experiments. Neither have analyses of functional diversity. Tools such as FUNGuild (Nguyen et al., 2016) have become common tools in assessing the functional diversity of fungal communities. Assessments of functional genes have also become an accessible tool for moving past diversity measures and understanding what activities microbes are capable of undertaking. Quantifying the totality of changes C deprivation may cause in soil structure, physicochemical factors, biomass, biodiversity, and functional diversity is critical to understanding the extent to which activities that remove soil C disrupt soil processes.

Here, we integrate such analyses to investigate the impact of long-term C deprivation on soil structure and biodiversity. Comparisons of soil structure, physicochemical properties, biomass, microorganismal diversity (specifically prokaryotes, fungi, and protists), and functional gene presence were made in a set of replicated plots, which comprised vegetated and bare (non-vegetated) areas established for 1 year and 10 years. Additionally, we identified bioindicators of vegetated and non-vegetated states within these treatments to demonstrate their effectiveness as markers of soil status following Paoletti’s (1999) definition of a bioindicator as an organism that is commensurate to the changes in their habitat. Our aim was to determine how C deprivation impacts soil structure, in order to meet this aim we set out to: (i) determine the consequences of long-term C depletion on soil structure; (ii) determine if long-term C depletion would cause a shift in functional and biological diversity; and (iii) identify taxa indicative of C deprived soils. Based on previously reported findings, we expected significant reductions in soil structure and biodiversity after long-term C depletion. Furthermore, we expected to observe an increase in functional genes involved in processing recalcitrant C.

## Materials and Methods

### Experimental Design

The experiment was established in 2005 at Bangor University’s Henfaes Research Centre, Abergwyngregyn, United Kingdom (53.24°N, 4.02°W; Elevation 12 m a.s.l.). Six 9 m^2^ plots were established on a Eutric Cambisol soil in a field previously used for sheep (*Ovis aries* L.) grazing. All six plots were fenced to exclude grazers and demarcated with plastic frames embedded 25 cm into the soil, with 5–8 cm protruding aboveground (Supplementary Figure 1A). Half (*n* = 3) of these plots were left vegetated and half (*n* = 3) covered (non-vegetated) by a thin layer of freely gas and water permeable black polypropylene landscaping anti-weed fabric for the duration of the experiment (Supplementary Figure 1B). Additions of polypropylene to this soil at concentrations of ≤0.1% (v/v) have shown no effect on the soil metabolome or microbiome over a 12-month period (Jones et al., unpublished) and no visible plastic was present in the soil at the time of sampling. We chose this approach over the traditional fallow approach, which uses herbicide and plowing to suppress plant growth, to minimize indirect effects on soil structure and the microbial community. These plots were maintained for 10 years. The vegetated plots were mown twice each year (May and September). In January 2015, a further eight plots (four vegetated and four non-vegetated) were established adjacent to the initial trial plots, within the same field (Supplementary Figure 1A) to investigate the early impacts of C deprivation. Vegetated plots were composed of a mixed grass community dominated by *Lolium perenne* L. with *Holcus lanatus* L. and *Festuca ovina* L. A total of 10 subsamples were collected, pooled, and homogenized on a per replicate basis in spring 2016 using 1 cm diameter soil corers at 10 cm depth.

### Soil Properties

Moisture content was calculated by oven drying. Electrical conductivity (EC; μS cm^–1^) and pH were measured using a 1:2.5 (w/v) dH_2_O extraction. Samples for total C (%) and N (%) analyses were air dried (20°C), milled to a <0.2 mm powder, and oxidized in a TruSpec CN Analyzer (LECO Corp., St. Joseph, MI, United States). Ammonium (NH_4_^+^) and nitrate (NO_3_^–^) were determined colorimetrically in a 1:5 (w/v) 0.5 M K_2_SO_4_ extraction (Mulvaney, 1996; Miranda et al., 2001). Available phosphorous (P) was also analyzed colorimetrically in a 1:5 (w/v) dH_2_O extraction (Murphy and Riley, 1967). These nutrients were all reported as mg kg^–1^. Cations (nmol kg^–1^) including potassium (K), sodium (Na), magnesium (Mg), and aluminum (Al) were obtained in a 1:5 (w/v) 1 M ammonium acetate extraction and analyzed using a 700 Series ICP-OES (Agilent Technologies, Inc., Santa Clara, CA, United States). Cation exchange capacity (CEC; mmol NH_4_^+^ kg^–1^) was calculated using ammonium acetate colorimetric methodology (Summer and Miller, 1996).

*In situ* CO_2_ flux measurements were recorded using an automated LI-8150 multiplexer CO_2_ flux system (LI-COR, Inc., Lincoln, NE, United States). Polyvinyl chloride collars were inserted ∼5 cm into the soil to house 20.3 cm diameter dark chambers (LI-COR LI-8100-104) in each plot. Soil CO_2_ flux was measured every ∼2 h over the course of 7 days in June 2015 (141 total measurements), with an automated infrared gas analyzer (LI-COR LI-8100) attached to the multiplexer system. Soil temperature was also recorded at this time.

### Soil Structure

Soil structure was assessed by soil porosity analyses at the University of Nottingham’s Hounsfield Facility. Undisturbed soil cores of 7 cm diameter and ∼16 cm depth were scanned using a v| tome| x M 240 kV X-ray Computed Tomography (μCT) scanner (GE Sensing & Inspection Technologies GmbH, Wunstorf, Germany) with an electron acceleration energy of 170 kV, current of 200 mA, and resolution of 40 μm. In total, 2,400 projection images were collected during each 140 min scan. Reconstruction was performed using Datos| Rec software (GE Sensing & Inspection Technologies GmbH, Wunstorf, Germany) and 2,272 images were collected for each sample. The data were then subsampled to cuboid volumes, sized 40 mm × 40 mm × 1700 images to avoid edge effects caused during sample collection or in the subsequent analysis. Pore space was separated from the surrounding soil matrix using the Li global automatic threshold algorithm (Li and Tam, 1998). Total porosity, pore size distribution, mean pore size, total pore area, and pore connectivity (expressed as the Euler number) were then analyzed using ImageJ software (Schneider et al., 2012). The coefficient of uniformity (a ratio of pore size distribution expressed by d_60_:d_10_) (Kézdi, 1974) was calculated as a simple way of expressing the pore size distribution.

Following initial scanning, cores were also cut into three equal parts (top, middle, and bottom) and air-dried for 2 days. An aggregate of approximately 4 mm diameter was randomly selected from each dried sample, with three aggregates per core collected representing each layer of the sample. Aggregates were scanned using a Phoenix Nanotom 180NF scanner (GE Sensing & Inspection Technologies GmbH, Wunstorf, Germany) with an electron acceleration energy of 90 kV, current of 70 mA, resolution of 3 μm, and 1,800 projection images being collected over 133 min. For analysis, scans were subsampled to a 1.4 mm × 1.4 mm × 600 slice cube from the center of each aggregate. Like the whole column scans, pore space was identified using the Li global automatic threshold algorithm and the same measurements taken. Based on the results from 10-year C deprived soils, we did not expect meaningful differences in the 1-year C deprivation soils and chose not to pursue these analyses for this treatment.

### Phospholipid Fatty Acid Analysis

Phospholipid fatty acid analyses (PLFA) were performed by Microbial ID, Inc. (Newark, DE, United States). In total, 10 g of sieved (2 mm) soil from each sample were frozen (−80°C), freeze-dried and sent for analysis on dry ice. Data generated included: total PLFA (nmol g^–1^), total bacteria and fungi, as well as *Actinobacteria*, anaerobes, Gram-positive, and Gram-negative bacteria. The microbial metabolic quotient (qCO_2_; μmol CO_2_ m^–2^ s^–1^_/_nmol g^–1^) was derived from total PLFA and soil CO_2_ flux measurements.

### DNA Extraction and Sequencing

DNA was extracted from 0.25 g of soil of each homogenized sample using a MO-BIO PowerLyzer PowerSoil DNA Isolation kit (Qiagen, Hilden, Germany). Amplicon libraries were created in triplicate and processed through a two-step library preparation protocol followed by Illumina MiSeq (San Diego, CA, United States) DNA sequencing at the Centre for Genomic Research, University of Liverpool. Primers used for the first round PCR were: 515F/806R (Caporaso et al., 2011; Walters et al., 2011), ITS5/5.8S_fungi (Epp et al., 2012), and TAReuk454FWD1/TAReukREV3 (Behnke et al., 2011) for the 16S rRNA, ITS1, and 18S rRNA gene libraries, respectively. Each PCR used 1 μL DNA (10 ng/μl concentration) and began at 98°C for 30 s and ended at 72°C for 10 min followed by a 4°C final extension for 10 min using a DNA Engine Tetrad^®^ 2 Peltier Thermal Cycler (Bio-Rad Laboratories, Inc., Hercules, CA, United States). First-round PCR amplification for the 16S rRNA gene library followed 10 cycles of 98°C for 10 s; 50°C for 30 s; 72°C for 30 s. For the ITS1 library there were 15 cycles of 98°C for 10 s; 58°C for 30 s; 72°C for 30 s. For the 18S rRNA gene library there were 15 cycles of 98°C for 10 s; 50°C 30 s; 72°C for 30 s. Next, 12 μl of each first-round PCR product were mixed with 0.1 μl of exonuclease I, 0.2 μl of thermosensitive alkaline phosphatase, and 0.7 μl of water. This mixture was cleaned in the thermocycler using a program of 37°C for 15 min and 74°C for 15 min. Illumina Nextera XT 384-way indexing primers were then added and amplified using a single protocol: initial denaturation at 98°C for 3 min; 15 cycles of 95°C for 30 s; 55°C for 30 s; 72°C for 30 s; and a final extension at 72°C for 5 min followed by a 4°C hold. Last, 25 μl of second-round PCR products were purified with 25 μl of AMPure XP beads (Beckman Coulter, Inc., Brea, CA, United States).

Bioinformatics were performed on the Supercomputing Wales system following George et al. (2019). Taxonomy was assigned using QIIME 1.9.1 (Caporaso et al., 2010) using default parameters with RDP methodology (Wang et al., 2007) using GreenGenes v. 13_8 (DeSantis et al., 2006) for the 16S OTU table, UNITE v. 7.2 (Kõljalg et al., 2013) for the ITS1 OTU table, and SILVA 128 (Quast et al., 2013) for the 18S OTU table. In addition, the ITS1 OTU table was passed through FUNGuild (Nguyen et al., 2016) to identify functional groups. Singletons were removed. Samples were rarefied 100 times for each OTU table using phyloseq (McMurdie and Holmes, 2013) and the rounded mean used for all analyses. All OTU tables were rarefied to depths of 19,311 reads for 16S rRNA gene sequences, 3,243 reads for ITS1 sequences, and 69,908 reads for 18S rRNA gene sequences. Sequences are available on the European Nucleotide Archive (Primary Accession Code: PRJEB33898).

### GeoChip Analyses

Soils from the 10-year treatment were selected for functional gene analyses. DNA from these plots analyzed using GeoChip (Glomics Inc., Norman, OK, United States). DNA extracts were fluorescently labeled with a cyanine dye, hybridized to GeoChip 4.0 microarrays for 16 h, and then non-binding DNA was washed away. Average signal intensity of fluorescently labeled DNA molecules bound to gene probes was measured with a laser scanner and standardized to remove background signal (signal-to-noise ratio <2). Genes involved in C degradation and anaerobic respiration, including N, S, and P cycling were studied.

### Statistical Analyses

The phyloseq package was used to calculate α-diversity. Within each C deprivation treatment, soil properties, PLFA ratios, OTU richness, and Shannon-Weiner index (H’) values from vegetated and non-vegetated soils were compared with two-tailed student’s *T*-tests in R 3.3.3 (R Core Team, 2017). Spearman’s correlations were assessed for richness with soil and environmental properties. Conical analyses of principle coordinates (CAP) were used to assess ordination of β-diversity and its relationship to soil and environmental properties for each subset of the community. Differences in β-diversity were assessed by PERMANOVA and homogeneity of variance tests. Pore morphology measurements were assessed by a one-tailed student’s *T*-test in R. Bioindicator taxa were selected using linear discriminant analyses (LDA) with the LDA Effect Size (LEFSe) method (Segata et al., 2011) with default parameters in Galaxy (Afgan et al., 2018). Due to the large number of prokaryotic and protistan bioindicators identified with this method, we present only those with the highest LDA scores (prokaryotes >4; protists >3.5). Similarly, OTU-level indicators were identified using differential abundance analyses from the R package DESeq2 (Love et al., 2014) on unrarefied OTU tables. Complete DESeq2 and LDA outputs are can be found in Supplementary Tables 4–6 and Supplementary Figures 5, 6. For GeoChip data, log-fold changes in signal frequency for genes of interest were identified. Again, student’s *T*-tests with Bonferroni correction were used to compare differences between treatments.

## Results

### Soil Properties

There were few significant differences between soil physicochemical properties in vegetated and non-vegetated soils after 1 year of C deprivation (Table 1). No differences were observed in pH, EC, NH_4_^+^, available P, Ca, K, Mg, CEC, total cations, C, N, or C: N ratio (Table 1). There was however, a significantly higher level of Na in vegetated soils (*P* < 0.001) and significantly lower moisture content in addition to concentration of NO_3_^–^ (*P* = 0.04). Soil CO_2_ flux was also higher in vegetated soils (*P* < 0.001; Table 1).

**TABLE 1:** Physicochemical properties from soils subjected to carbon deprivation for 1 year and 10 years.

Soil physicochemical properties	1 year		10 years	
	Vegetated	Non-vegetated	Vegetated	Non-vegetated
pH	5.04 (±0.05)	4.79 (±0.04)	5.46 (±0.04)*	4.67 (±0.06)
Electrical conductivity (μS cm^–1^)	118 (±7.5)	207 (±26.3)	104.7 (±2.2)	149.3 (±2.9)*
Soil CO_2_ flux (μmol m^–2^ s^–1^)	6.85 (±0.58)***	2.04 (±0.22)	4.97 (±0.29)***	0.99 (±0.24)
Moisture (% dry weight)	23.9 (±0.5)	43.1 (±2.8)***	35.8 (±1.5)	34.4 (±0.1)
Total C (%)	3.11 (±0.16)	3.57 (±0.18)	3.73 (±0.17)*	2.54 (±0.34)
Total N (%)	0.31 (±0.01)	0.34 (±0.01)	0.32 (±0.01)*	0.23 (±0.02)
C: N ratio	9.96 (±0.16)	10.39 (±0.36)	11.81 (±0.28)	10.75 (±0.56)
Nitrate (mg NO_3_^–^ kg^–1^)	2.09 (±0.53)	30.45 (±10.49)*	0.88 (±0.19)	0.35 (±0.12)
Ammonium (mg NH_4_^+^ kg^–1^)	43.51 (±10.93)	38.01 (±9.96)	1.29 (±0.14)	1.86 (±1.14)
Available Phosphorus (mg P kg^–1^)	3.36 (±0.30)	3.28 (±0.09)	4.46 (±0.68)	3.21 (±1.05)
Calcium (nmol Ca kg^–1^)	19.78 (±1.59)	18.76 (±0.71)	18.28 ± (2.91)**	4.25 (± 0.77)
Potassium (nmol K kg^–1^)	2.77 (±0.20)	4.10 (±0.34)	0.82 (±0.07)*	0.54 (±0.03)
Sodium (nmol Na kg^–1^)	0.96 (±0.07)**	0.61 (±0.19)	6.48 (±0.65)**	0.99 (±0.07)
Magnesium (nmol Mn kg^–1^)	4.28 (±0.29)	3.86 (±0.16)	0.01 (±5.3 × 10^4^)***	0.004 (±3.86 × 10^–4^)
Aluminum (nmol Al kg^–1^)	N.D.	N.D.	0.001 (±0.001)	0.03 (±0.003)***
Total cations (nmol kg^–1^)	48.52 (±3.49)	43.09 (±1.36)	43.89 (±6.31)**	10.45 (±1.47)
Cation exchange capacity (nmol kg^–1^)	974.48 (±29.40)	917.75 (±49.10)	56.68 (±4.52)*	40.80 (±3.22)
Temperature (°C)	15.3 (±0.1)	15.6 (±0.1)	15.1 (±0.1)	16.4 (±0.2)**

Mean values (±SE) are presented for both treatments within each age class. Significantly greater values within each age class are indicated by: ***P < 0.001, **P < 0.01, *P < 0.05, or blank (*P* > 0.05). Aluminum was not determined (N.D.) in 1 year-old samples.

After 10 years of C deprivation, differences in physicochemical properties between the vegetated and non-vegetated soils were more prevalent. Total C (*P* = 0.04) and N (*P* = 0.03) were also significantly greater in vegetated soils, though C: N did not change (Table 1). Both pH and EC (both *P* = 0.01) were significantly different between vegetated and non-vegetated soils, with lower pH and higher EC in the absence of plants. Concentrations of all cations, except Al and Mg, and CEC as well as soil CO_2_ flux were significantly greater in vegetated soils. Interestingly, Al and Mg were significantly higher in the non-vegetated soils (*P* < 0.001). Temperature was significantly greater (*P* = 0.003) in the non-vegetated plots.

### Soil Pore Analyses

**FIGURE 1:**
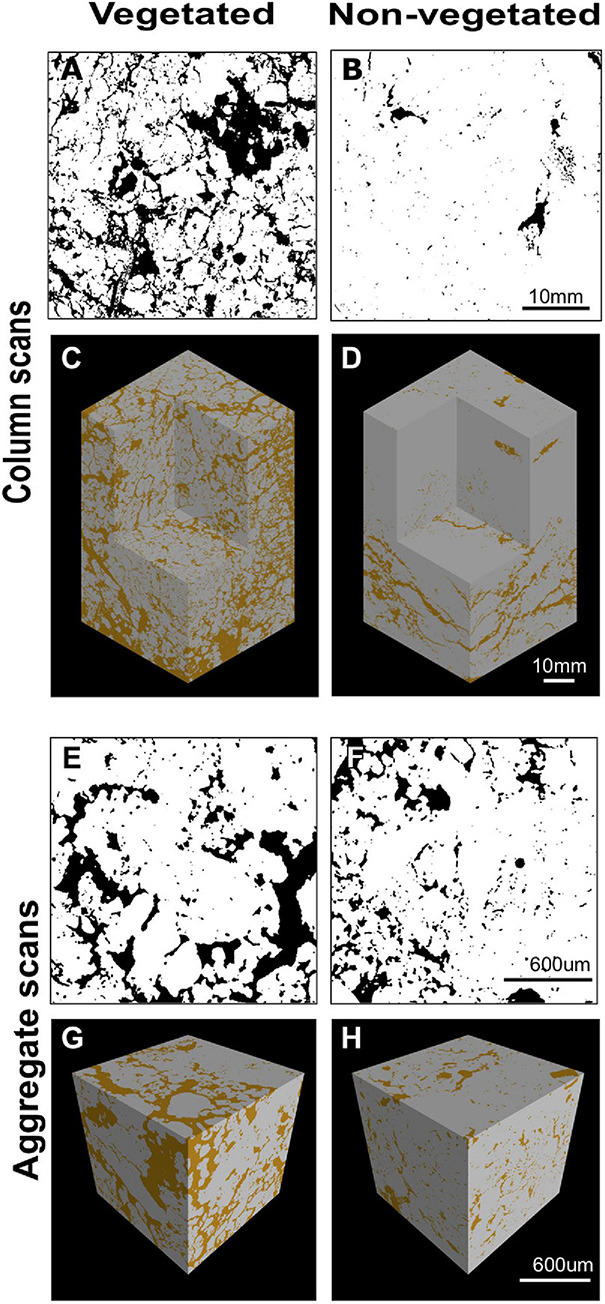
Example soil porosity images collected using x-ray μCT. 2D binary images collected from the center of a vegetated **(A)** or non-vegetated **(B)** column. Pore space shown in black, soil in white. 3D reconstruction of soil columns from vegetated **(C)** and non-vegetated **(D)** columns. 2D binary images collected from the center of a vegetated **(E)** or non-vegetated **(F)** aggregate. Pore space shown in black, soil in white. 3D reconstruction of soil aggregates from vegetated **(G)** and non-vegetated **(H)** aggregates. Pore space shown in brown, soil in gray.

X-ray CT imaging revealed clear differences in soil structure between 10-year non-vegetated and vegetated soils (Figure 1). We present 3D images of pore architecture from the vegetated and non-vegetated soils at the column (Figures 1C,D) and aggregate (Figures 1G,H) scales. In general, the vegetated soils appear more porous with large pores that were more connected, though this was more apparent at the column scale than the aggregate scale. Pore morphology measurements revealed a decline of all porosity characteristics in non-vegetated soils – note that a large Euler number indicates reduced pore connectivity (Supplementary Table 1). At the column scale, total porosity and total pore area of the non-vegetated soils were lower (Figures 1A,B) than vegetated soils though not statistically significant (both: *P* = 0.07). However, mean pore size was significantly reduced in the absence of plant inputs (*P* < 0.001). Pore size distribution (Supplementary Figure 2A) showed a reduction in the size of pores across all classes in the non-vegetated in comparison with the vegetated soils, with a greater reduction generally in the number of larger pore size classes. This is demonstrated by the coefficient of uniformity value of c.110 in the vegetated soil versus c.58 in the 10-year non-vegetated treatment.

A similar pattern was observed at the aggregate-level, with total porosity and pore area being reduced in the non-vegetated samples (Figures 1E,F), although to a lesser extent than at column scale (*P* = 0.25 and *P* = 0.22, respectively). Again, mean pore size was significantly lower in the non-vegetated soils (*P* < 0.001) with a mean value of 0.05 mm^2^ in the vegetated soil compared to 0.01 mm^2^ in the non-vegetated soil. Pore size distributions were similar between non-vegetated and vegetated treatments at the aggregate scale in the smaller pore size classes, except for in larger pore size classes where more large pores were recorded in the vegetated plots (Supplementary Figure 2B). Interestingly, at the aggregate scale there was higher pore connectivity recorded in the 10-year non-vegetated plots (observed as a higher Euler number) though this was not significantly different (Supplementary Table 1).

### Microbial Biomass

Trends in microbial biomass inferred from PLFA analysis were broadly similar across both C deprivation treatments (Table 2). After 1-year C deprivation, total PLFA biomass, the proportions of fungal (*P* < 0.001) and bacterial (*P* = 0.02) biomass, and the fungi: bacteria ratio (*P* < 0.001), were all significantly greater in the presence of plants (Table 2). The proportion of anaerobe biomass in these soils did not meet detection thresholds. After 10 years of C deprivation, total PLFA biomass (*P* = 0.03) and the fungi: bacteria PLFA ratio (*P* < 0.001) were significantly greater in vegetated soils (Table 2). When assessed by proportional contribution to the total PLFA biomass, the proportions of fungi (*P* = 0.002) and anaerobes were significantly greater (*P* = 0.01) in vegetated sites, though proportions of Gram-positive bacteria, *Actinobacteria* (both *P* = 0.03), and total bacteria (*P* < 0.001) were higher in non-vegetated soil (Table 2). The qCO_2_ was significantly greater in vegetated soils in both the 1- and 10-year experiments (*P* < 0.001, *P* = 0.01, respectively).

**TABLE 2:** Microbial biomass fractions from phospholipid fatty acid analysis (PLFA) from soils subjected to carbon deprivation for 1 year and 10 years.

Microbial biomass from PLFA analyses				
	1 year		10 years	
	Vegetated	Non-vegetated	Vegetated	Non-vegetated
Total PLFA (nmol g^–1^)	246.0 (±7.9)*	212.2 (±3.0)	190.8 (±24.5)**	107.8 (±6.2)
Gram-negative (%)	43.1 (±0.2)	43.0 (±0.3)	48.7 (±0.2)	47.8 (±0.5)
Gram-positive (%)	29.7 (±1.7)	37.8 (±2.0)	26.6 (±0.2)	28.3 (±0.3)**
*Actinobacteria* (%)	11.17 (±1.78)	10.73 (±2.08)	13.53 (±0.22)	15.33 (±0.47)*
Anaerobes (%)	N.D.	N.D.	1.04 (±0.02)*	0.81 (±0.08)
Fungi (%)	6.35 (±0.11)**	4.85 (±0.11)	7.17 (±0.3)**	5.19 (±0.21)
Bacteria (%)	91.59 (±0.16)*	90.03 (±0.20)	89.83 (±0.31)	92.23 (±0.42)**
Fungi: bacteria ratio	0.08 (±0.002)**	0.06 (±0.001)	0.10 (±0.004)***	0.07 (±0.003)
qCO_2_ (μmol CO_2_ m^–2^ s^–1^_/_nmol g^–1^)	0.03 (±0.002)***	0.001 (±0.001)	0.03 (±0.003)*	0.01 (±0.003)

Mean values (±SE) are presented for both treatments within each age class. Significantly greater values within each age class are indicated by: ***P < 0.001, **P < 0.01, *P < 0.05, or blank (*P* > 0.05). Percentage of anaerobes was not determined (N.D.) in 1 year-old samples.

### Microbial Diversity Metrics

**FIGURE 2:**
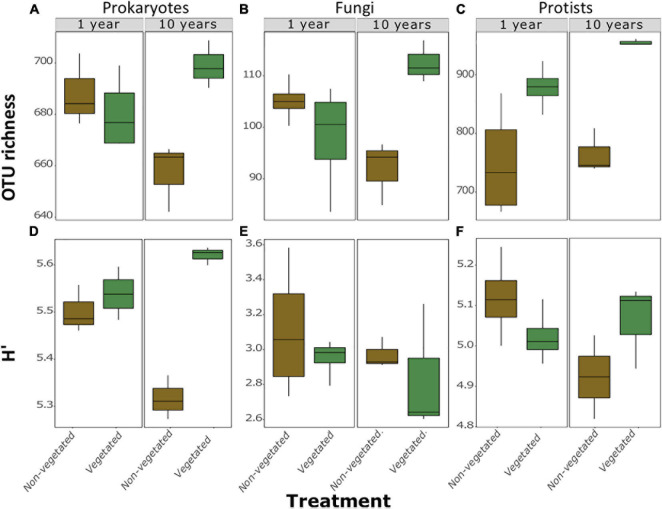
Measures of α-diversity of soil community fractions in non-vegetated and vegetated soils. Each plot is split by age. Plots **(A–C)** show richness of bacteria, fungi, and protists, respectively. Plots **(D–F)** show Shannon-Weiner diversity (H’) values of bacteria, fungi, and protists, respectively. No significant differences were observed in the 1 year treatment for either metric or for H’ of fungi and protists after 10 years of C deprivation.

A total of 973 prokaryotic, 336 fungal, and 1,638 protistan OTUs were identified across all samples. There were no significant differences between richness or H’ between vegetated and non-vegetated treatments in 1-year-old sites across prokaryotes, fungi, or protists (Figure 2). However, richness of prokaryotes, fungi, and protists was lower in the 10-year-old non-vegetated treatment (Figures 2A–C; all *P* = 0.01). This was also the case for H’ values for prokaryotes (Figure 2D; *P* < 0.003) but not for fungi (Figure 2E; *P* = 0.59) or protists (Figure 2F; *P* = 0.17).

Our CAP ordinations demonstrated that there were significant differences in β-diversity between treatments. There was a significant (*F*_3_,_9_ = 5.7, *P* = 0.001) difference between prokaryotic communities, likely driven by differences in the 10-year treatments, though 1-year treatments were closely nested together. This relationship became less clear for fungi and protists, though there were significant (*F*_3_,_10_ = 3.34, *P* = 0.001; *F*_3_,_10_ = 4.74, *P* = 0.001; for fungi and protists, respectively) differences here too, again likely due to the severe reduction in biodiversity in the 10-year vegetated plots.

Relationships between soil and environmental factors and diversity revealed group-specific relationships. All of these interactions showed strong relationships with the vegetated treatments. Indeed, CAP ordinations show all significant variables trend away from the 10-year old non-vegetated treatment (Supplementary Figure 3). Metrics of α-diversity showed similar trends (Supplementary Tables 2, 3). Richness (Supplementary Table 2) of prokaryotes and protists were significantly positively correlated with pH (*P* = 0.01; *P* = 0.001, respectively; Supplementary Table 2). Total C had strong positive correlations with fungi (*P* = 0.006) and prokaryotes (*P* = 0.003). Protists also had a significant, negative, relationship with EC (*P* = 0.002) and positive relationships with pH (*P* = 0.001), and Na (*p* = 0.008). There were more pronounced trends in H’, with H’ of prokaryotes displaying strong positive correlations with pH, Ca, total cations (all *P* < 0.001), total C (*P* = 0.008), N (*P* = 0.009), and CO_2_ flux (*P* = 0.005). Protistan H’ had significant positive relationships with total C (*P* = 0.02) and total N (*P* = 0.01). There were no significant correlations between fungal H’ (Supplementary Table 3).

### Changes in Function

Fungal functional diversity showed strong similarities between both sets of vegetated plots. There was a marked increase in OTUs suspected to have pathotrophic or saprotrophic trophic modes in the 1-year non-vegetated plots. There was a noticeable proportion of symbiotrophs in the 10-year non-vegetated treatment, likely a consequence of the very low OTU richness in these samples (Supplementary Figure 4).

**FIGURE 3:**
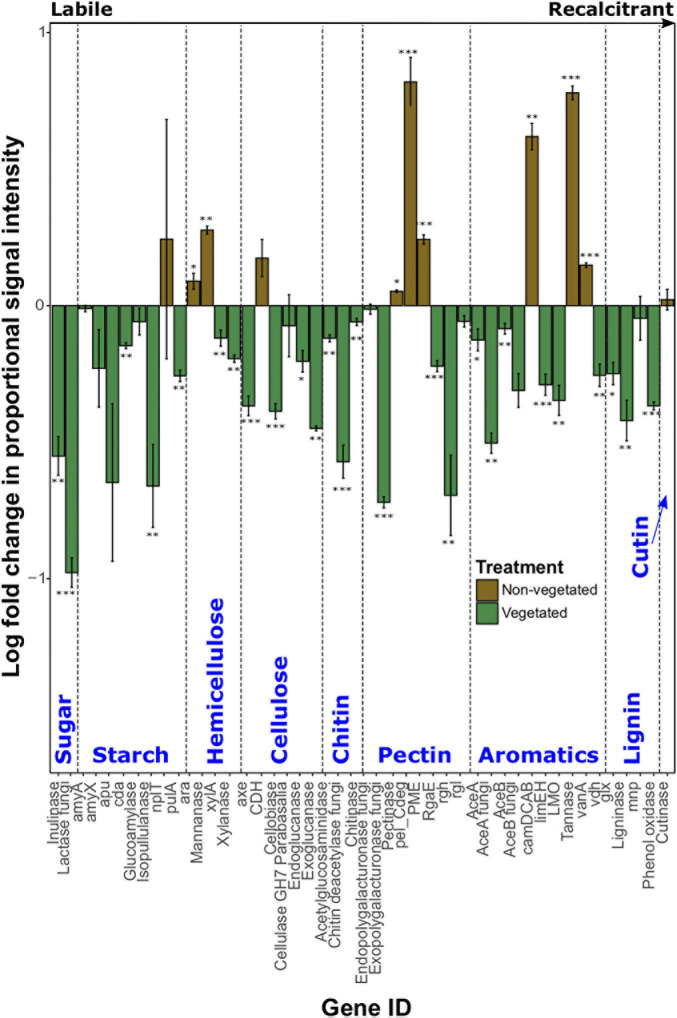
Logarithmically transformed fold change in signal intensity from GeoChip data of carbon degradation genes. Genes are ordered from labile to recalcitrant. Error bars denote standard error. Significant differences are indicated by: ^∗∗∗^*P* < 0.001, ^∗∗^*P* < 0.01, ^∗^*P* < 0.05, or blank (*P* > 0.05).

Log-fold change of most C degradation genes (55.3%) was significantly greater (*P* < 0.05) in vegetated soils than non-vegetated C-deprived soils (Figure 3). These included more labile C forms (sugar, starch, and cellulose) and the majority of genes involved in degrading more recalcitrant or complex C forms. Yet in the 10-year-old treatments there was a significant increase in proportional signal intensity under non-vegetated conditions for eight genes that are involved in the degradation of recalcitrant C, including pectin (pectinase, pel_Cdeg, and PME) and aromatics (camDCAB, tannase, and vanA). There was also a significant increase in the proportion of hemicellulose degrading genes like the L-arabinose operon (*P* = 0.02) and mannanase (*P* = 0.001) (Figure 3). Of note, variation of signal intensities for some genes (i.e., pulA and apu) meant that significant differences could not be identified, despite clear differences in absolute values (Figure 3). Data from fungal xylose reductase are omitted from Figure 3 as it did not meet the criteria for analyses in all non-vegetated samples, but was present in all vegetated samples (*P* < 0.001).

**FIGURE 4:**
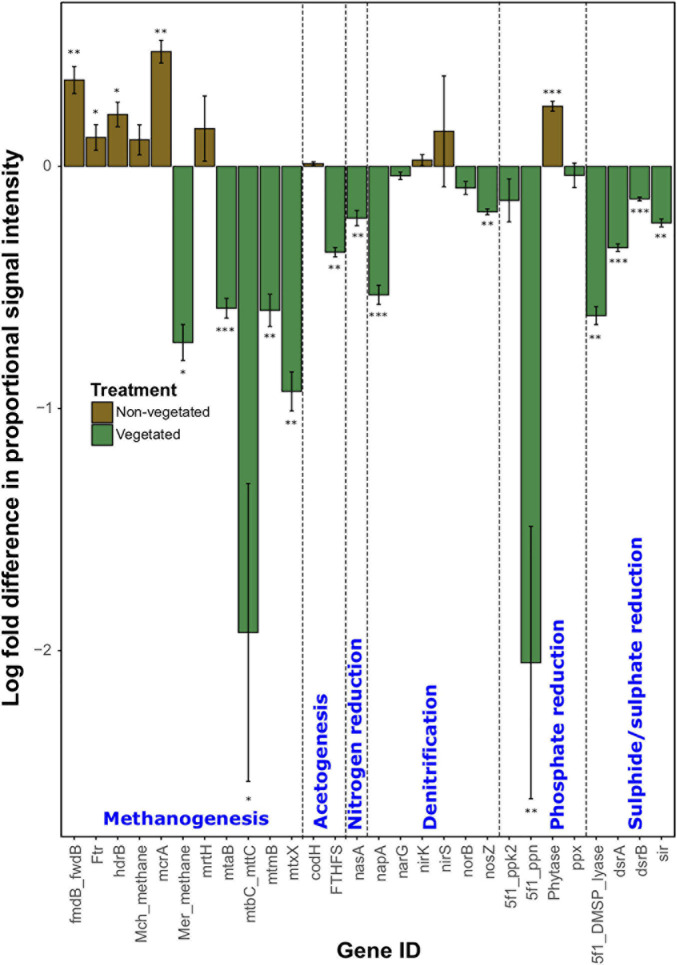
Logarithmically transformed fold change in signal intensity from GeoChip data of anaerobic functional genes. Error bars denote standard error. Significant differences are indicated by ^∗∗∗^*P* < 0.001, ^∗∗^*P* < 0.01, ^∗^*P* < 0.05, or blank (*P* > 0.05).

As with C degradation genes, the log-fold change of the majority (61.1%) of genes involved in anaerobic respiration processes were significantly (*P* < 0.05) more abundant in vegetated samples (Figure 4). This included eight genes (*ACS, cdhC_methane, mtmC, mttB, Hmd, mtaC, mtbB*, and *MT2*) involved in methanogenesis. Although significantly greater in vegetated soils (*P* < 0.001), these genes were omitted from Figure 4 as they did not meet detection thresholds in non-vegetated plots. Other methanogenesis genes showed a differential response to treatments. For example, methyltransferases (*mtaB*, *mtbC_mttC*, *mtmB*, and *mtxX*) had greater log-fold presence in vegetated soils, whereas four genes involved in methanogenesis (*fmdB_fwdB*, *Ftr*, *hdrB*, and *mcrA*) had greater presence in non-vegetated soils. Phytase also showed an increased log-fold change in non-vegetated soils (Figure 4). Certain genes involved in acetogenesis (*FTHFS*), N cycling (*nasA*, *napA*, and *nosZ*), and the reduction of phosphate and sulfur compounds also increased in abundance in vegetated soils (Figure 4).

### Identification of Bioindicator Taxa for Vegetated and Non-vegetated Soils

**FIGURE 5:**
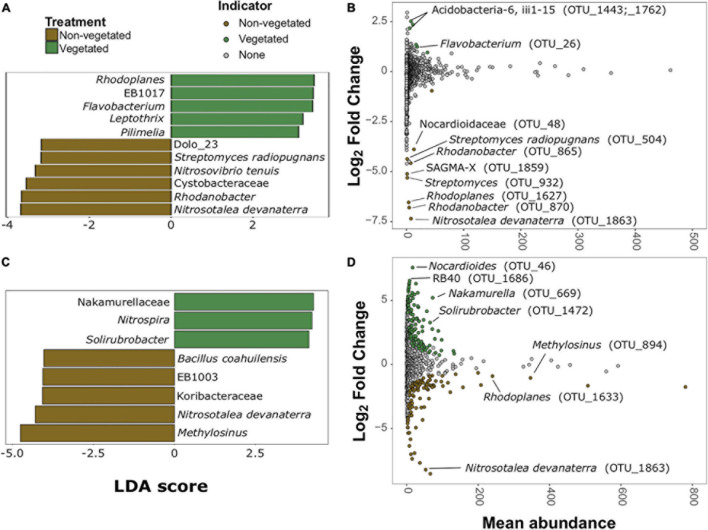
Prokaryotic bioindicators of non-vegetated and vegetated soils. Indicator taxa identified by linear discriminant analyses (LDA) with scores greater than 4 for **(A)** 1-year and **(C)** 10-year C deprivation. OTUs indicative of treatment based on corrected *P* values from DESeq2 analysis identified in **(B)** 1-year and **(D)** 10-year C deprivation.

There were more prokaryotic indicator taxa in soils from the 10-year treatment than the 1-year treatment (Figures 5A,C). In the 1-year dataset there were 11 indicator taxa, of which, five were characteristic of vegetated and six of non-vegetated soils (Figure 5A). *Rhodoplanes* (LDA 3.5) and *Flavobacterium* (LDA 3.47) were the best bacterial indicator taxa of vegetated 1-year-old sites. *Nitrosotalea devanaterra* (LDA 3.68) and *Rhodanobacter* (LDA 3.66) were the best indicators for 1-year C deprivation non-vegetated sites (Figure 5A). *Nakamurellaceae* (LDA 4.29), *Nitrospira* (LDA 4.25), and *Solirubrobacter* (LDA 4.15) were the best indicators of vegetated soils after 10 years. *Methylosinus* (LDA 4.75) and *N. devanaterra* (LDA 4.3) were the strongest indicators of 10-year C deprived non-vegetated (Figure 5C).

Identification of differentially abundant OTUs through DESeq2 also highlighted *N. devanaterra* (OTU_1863) and *Rhodanobacter* OTUs (OTU_865 and OTU_870) indicators in 1-year C deprived non-vegetated (Figure 5B). Also, OTUs identified as *Streptomyces* (OTU_932 and OTU_504), were more abundant in 1-year non-vegetated soils (Figures 5A,B). In 10-year C deprived soils, an OTU identified as *Solirubrobacter* (OTU_1472), was noted as an indicator of vegetated soil and *Paenibacillus* was highlighted as an indicator of non-vegetated soils (Figure 5D), which support our LDA results (Figures 5C,D and Supplementary Figure 5). Unexpectedly, some differentially abundant OTUs matched to indicator taxa from opposing treatments. For example, *Rhodoplanes* was an indicator of vegetated soil in LDA data from the 1-year treatment (Figure 5A) but some OTUs (OTU_1627 and OTU_1633) were more abundant in both treatments’ non-vegetated soils (Figures 5B,D and Supplementary Figure 5).

**FIGURE 6:**
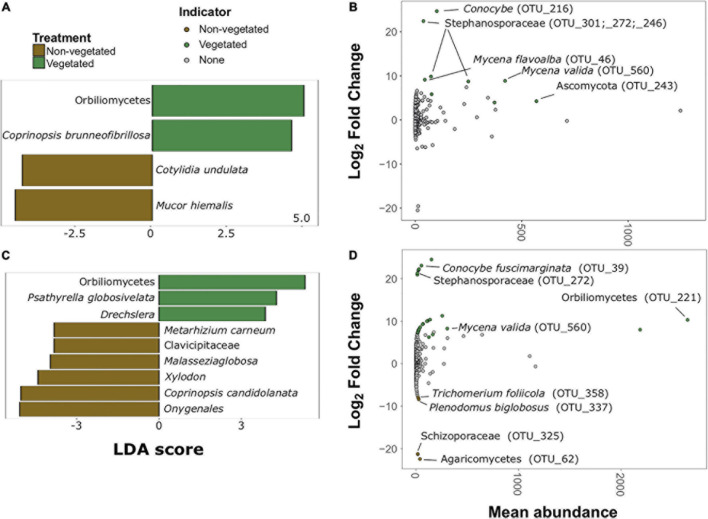
Fungal bioindicators of non-vegetated and vegetated soils. Indicator taxa identified by linear discriminant analyses (LDA) for **(A)** 1-year and **(C)** 10-year C deprivation. OTUs indicative of treatment based on corrected *P* values from DESeq2 analysis identified in **(B)** 1-year and **(D)** 10-year C deprivation.

There were much fewer fungal indicator taxa and differentially abundant OTUs for both C deprivation treatments (Figure 6). Only four indicator taxa (two vegetated and two non-vegetated) from the 1-year treatment and nine indicator taxa (three vegetated and six non-vegetated) were identified from the 10 year treatment (Figures 6A,C). Orbiliomycetes (LDA 4.79) and *Coprinopsis brunneofibrillosa* (LDA 3.97) were indicative of the vegetated soils and *Cotylidia undulata* (LDA 4.56) and *Mucor hiemalis* (LDA 4.22) were indicative of non-vegetated after 1-year of C deprivation (Figure 6A). Orbiliomycetes (LDA 5.55) was also the strongest indicator of vegetated soils after 10 years, whereas, *Onygenales* (LDA 5.28) and *Coprinopsis candidolanata* (LDA 5.27) were the strongest indicators of non-vegetated (Figure 6C).

Fungal OTUs with differentially abundant read counts in vegetated soils assessed with DESeq2 analysis included those identified as *Stephanosporaceae* (OTU_301, OTU_272, and OTU_246), two different *Mycena* species (OTU_46 and OTU_560), *Conocyb*e, (OTU_216) and an unnamed Ascomycota. There were no differentially abundant OTUs for non-vegetated soils after 1-year of C deprivation (Figure 6B). We found OTUs, identified as Orbiliomycetes (OTU_221), and *Conocyb*e *fuscimarginata* (OTU_39) were indicators of vegetated soils in the 10-year treatment (Figures 6C,D). OTUs indicative of 10 years non-vegetated included *Trichomerium foliicola* (OTU_358) and *Plenodomus biglobosus* (OTU_337) (Figure 6D).

**FIGURE 7:**
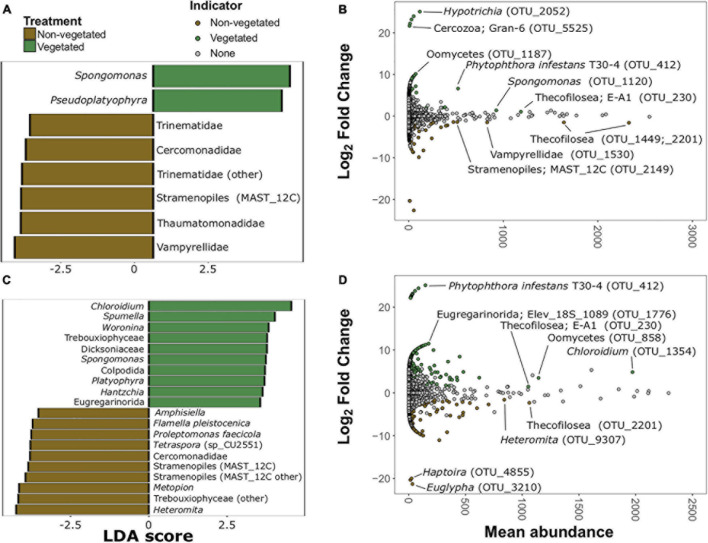
Protistan bioindicators of non-vegetated and vegetated soils. Indicator taxa identified by linear discriminant analyses (LDA) with scores greater than 3.5 for **(A)** 1-year and **(C)** 10-year C deprivation. OTUs indicative of treatment based on corrected *P* values from DESeq2 analysis identified in **(B)** 1-year and **(D)** 10-year C deprivation.

There were 20 protistan indicator taxa (nine vegetated and 11 non-vegetated) for 1-year C deprived soils (Figure 7 and Supplementary Figure 6) from LDA analysis. *Spongomonas* (LDA 4.07) was the strongest protistan indicator of vegetated soils in this treatment (Figure 7A). The families Vampyrellidae (LDA 4.12) and Thaumatomonadidae (LDA 3.94; Figure 7A) as well as various ambiguously identified cercozoans and Stramenopiles (Supplementary Figure 6A) were indicative of non-vegetated. In the 10-year C deprived treatment, there were 60 taxa indicative of vegetated soils and 29 taxa indicative of non-vegetated soils (Figure 7C and Supplementary Figure 6B). *Chloroidium* (LDA 4.45) and *Spumella* (LDA 4.03) were the strongest indicators of vegetated soils in this treatment (Figure 7C). Cercomonads, including *Heteromita* (LDA 4.63), Trebouxiophyceae (green algae; LDA 4.51), and multiple MAST_12C group Stramenopiles were characteristic of non-vegetated soils (Figure 7C).

*Spongomonas* (OTU_1120) and *Hypotrichia* (OTU_2052) were also indicative of vegetated soils in the 1-year treatment (Figure 7B). Similarly, OTUs belonging to Vampyrellidae (OTU_1530) and MAST_12C group Stramenopiles appear as indicators of the non-vegetated soils after 1 year of C deprivation (Figure 7B). *Heteromita* (OTU_9307) and members of the order Euglyphida were indicative of non-vegetated soils deprived of C for 10 years (Figure 7D), whereas *Chloroidium* and several Oomycetes were indicative of vegetated soils in this treatment.

## Discussion

### Effects of Long-Term C Deprivation on Soil Properties, Structure, and Biota

After 10 years of C deprivation, soils became more acidic and presented expected losses in C and N due to a lack of organic matter or plant-associated microbial necromass (Gregory et al., 2016; Hirsch et al., 2017). This is supported by the identification of *N. devanaterra* as a bioindicator of non-vegetated soils. *N. devanaterra* is an acidophile and although the pH range of all treatments is within the optimal range for *N. devanaterra* (Lehtovirta-Morley et al., 2011), its prevalence in the more acidic C deprived soils may indicate a shift to more favorable physicochemical conditions allowed these archaea to outcompete competitors.

Our data show a loss of approximately 0.12% C per year over the decade of treatment; although this is somewhat lower than 0.6% annual C loss expected for England and Wales (Bellamy et al., 2005), our work focused only on a Eutric Cambisol with an extensive agricultural history, whereas the estimate of Bellamy et al. (2005) accounts for a wide range of soil types. Long-term monitoring data from the UKCEH Countryside Survey also shows variability in C loss across the wider range of British soils (Reynolds et al., 2013), but confirms the significant loss in arable soils. In our study, there was no change in C: N ratio, suggesting that both elements were lost at a similar rate and did not cause a stoichiometric imbalance in organic matter. The acidifying effects of C deprivation were also expected, due to an increase in acidic cations (Paradelo et al., 2013). Carbon deprivation for only 1 year had markedly less of an effect on soil properties. Increased moisture retention in the smaller pool of organic matter under C deprivation (McGuire et al., 1998) may have caused leaching of Na and loss of NO_3_^–^ though being drawn into the topsoil via capillary action under moist conditions (Simpson, 1960).

Soils deprived of C for 10-years were characterized by reduced porosity, pore size, and pore connectivity at the column scale (40 μm). However, at higher spatial resolutions (3 μm), the differences between vegetated and non-vegetated were less pronounced, especially for total porosity and pore connectivity, which was actually greater (i.e., more negative) in non-vegetated soils than vegetated soils. These findings are consistent with Bacq-Labreuil et al. (2018), who found that connectivity of pores <0.09 mm in fallow soils was greater than in grassland soils with high sand content. Recently Neal et al. (2020) found similar results and revealed this enhanced pore connectivity has important implications for soil functions such as hydraulic conductivity. This trend toward increasing pore connectivity was surprising since we expected reduced structural development in the absence of plants for example, as pore connectivity has been previously shown to increase with increasing complexity of plant communities (Kravchenko et al., 2019). Our incongruent findings could be due to vestiges of macropores that have previously been lost due to biodegradation of labile organic matter lattices in non-vegetated soils over time (Xie et al., 2018) or linked to the activity of the faunal communities that existed under these conditions.

Although we observed an expected reduction in PLFA signals with C deprivation (Asuming-Brempong et al., 2008; Hirsch et al., 2009, 2017; Wu et al., 2012), our findings on microbial richness were contradictory to expected results. Hirsch et al. (2009, 2017) posited that soil biodiversity is resilient to 50 years of bare fallow. Yet we found that richness of bacteria, fungi, and protists all fell after 10 years in the absence of plant C inputs. Such a reduction in species richness indicates collapse of all fractions of the soil microbiosphere, despite increasing pore connectivity. Removal of vegetation leads to decreased richness of pathogens (Hirsch et al., 2017; Santos et al., 2020), symbionts (el Zahar Haichar et al., 2014; Hirsch et al., 2017), and saprotrophs (Hirsch et al., 2009, 2017; Wu et al., 2012) that rely on living plants and their detritus. We observed that these groups remained relatively consistent under C deprivation, and the proportion of fungal OTUs identified as potential pathotrophs actually increased after 1 year of C deprivation, perhaps exploiting the last dying or recently deceased plants. Additionally, vegetation removal also results in the loss of root exudates, which are major C sources and strong influencers of soil microbial community composition (el Zahar Haichar et al., 2014).

We did not observe the reduction in H’ of protists or fungi in 10-year C deprived non-vegetated soils that occurred for prokaryotes. Eukaryote diversity may be more resistant to C-deprived conditions because these organisms have a greater capacity to utilize multiple C-sources (Adl, 2003), employ resistance strategies to wait for ideal conditions, or simply posses greater motility to reach more distant food sources (Geisen et al., 2018). Indeed, most protistan bioindicators in the non-vegetated soils were likely motile consumers including various Cermonads, and other flagellates such as *Thecofilosea* and *Metopoin*, testate amoebae (*Euglypha*), and Haptorian ciliates. Changes in β-diversity were likewise driven by the significant losses in biodiversity under C-deprivation, though here they showed the opposite trend, with greater separation in the eukaryotic fractions after only 1 year of C deprivation. This suggests that each plot may possess eukaryotic communities with distinct compositions.

We also found evidence that fungi may be outcompeted by other organisms under C deprivation. There was a greater proportional abundance of bacterial biomass in 10-year C deprived non-vegetated soils driven by relative increases in Gram-positive bacteria and *Actinobacteria*. *Actinobacteria* occupy a similar niche to saprotrophic fungi exhibiting filamentous growth and many species utilize cellulose and lignin (de Boer et al., 2005). *Streptomyces* was identified as a bioindicator of non-vegetated soils, and is known to produce antifungal compounds (Doumbou et al., 2005), possibly contributing to the exclusion of fungi in non-vegetated soils. Another interesting bioindicator of 1-year-non-vegetated soils was Vampyrellid amoebae, which are predators of fungi (Hess et al., 2012), suggesting that there may be a shift in food webs deprived of C at this early stage. The spike and then marked reduction in plant-associated trophic groups, in the 1- and 10-year non-vegetated soils, specifically pathogens, suggests that plant resources have been exhausted.

Reduction of plant resources is further reinforced by the scarcity of fungal bioindicators we were able to identify compared to other microorganismal groups. Indeed, fungal indicator OTUs of C deprived soils had less certain affinities which could not be investigated in detail or belong to genera that may be exploiting local nutrient hotspots, such as NH_4_^+^ and low molecular weight organic N in the case of *Coprinopsis* (Raut et al., 2011). All other fungal bioindicators, such as Orbiliomycetes, representatives of the Stephanosporaceae, *Conocybe*, and *Mycena*, were indicative of vegetated soils. Similarly, Protistan bioindicators of vegetated treatments included plant pathogens, such as *Phytophthora* and other Oomycetes as well as Thecofilisean flagellates and the green algae *Chloroidium*.

### Effects of Long-Term C Deprivation on Soil Functional Diversity

After 10 years of C deprivation there was an increased presence of certain genes involved in methanogenesis and the degradation of more recalcitrant C forms. The increased proportion of certain methanogenesis genes is likely the result of an increase in methanogens in anaerobic microhabitats due to a loss of soil structure. Smaller pores, like those in our 10-year C deprived non-vegetated soils, generally experience more prolonged anoxia (Keiluweit et al., 2017). There was also an increase in proportion of the methyl coenzyme M reductase (*mcrA*) gene in non-vegetated soils, which is a marker gene for methanogens (Morris et al., 2014). Furthermore, we identified the methanotroph *Methylosinus* (Matsen et al., 2013) as a strong bioindicator of 10-year C-deprived soils. It is important to note that other methanogenesis genes, such as various methyltransferases (*mtaB*, *mtbC_mttC*, *mtmB*, and *mtxX*) were more abundant in vegetated soils. This inconsistency may indicate that although there was an increase in anaerobic conditions under C deprivation, vegetated soils also provide suitable conditions for methanogenesis. As the vegetated soils had larger, more connected pores, it is possible that an increase in water-filled pores (Keiluweit et al., 2017) promoted methanogen communities by reducing local redox potential (Dalal et al., 2008). Although we detected an increase in acetogenesis genes under non-vegetated conditions, we could not detect concurrent changes in archaeal biomass or biodiversity, which could cause an increase in aceticlastic methane production (Conrad, 2020).

The proportionally greater abundance of genes involved in hemicellulose, pectin, and aromatic degradation in the non-vegetated treatment suggests that the soil community has been exploiting some of the more complex plant biomass after 10 years without plant C inputs. Furthermore, our results show the absence of accessible low molecular weight C compounds, such as root exudates (Renella et al., 2006), under long-term non-vegetated may stimulate the release of C from more recalcitrant sources, potentially increasing CO_2_ emission rates (Lal, 2004).

Phytase was the only other gene that showed greater prevalence in non-vegetated soils (Figure 4), which parallels the reduction, though non-significant, of available P in non-vegetated soils (Table 1). This suggests that the anaerobic community has been forced to access poorly available P forms, specifically phytic acid (IP6), that is commonly found in soils (Richardson and Hadobas, 1997). Together, the data suggests that microbial communities in the absence of plants increase utilization of recalcitrant C energy sources in the absence of fresh organic matter inputs.

## Conclusion

We have shown that C deprivation of only a decade can reduce soil biodiversity measures, in contrast to previous work that found more resilience in the microbial community. We found that unexpectedly, pore connectivity and total porosity increased at higher resolutions of soil structure, despite reductions at lower scales. These seemingly incongruent results may be evidence of extensive C consumption as microorganisms exploit any remaining food sources. The overall reduction in microbial biomass and α-diversity after 10-years of C deprivation supports the notion that long-term soil C removal has significant detrimental impacts on microbial community composition and functioning, This is especially true of fungi, which appeared to be outcompeted by bacteria in the absence of fresh C inputs. Similarly, the relative increase in certain recalcitrant C and IP6 degrading genes suggests that the microbial community has exhausted the majority of low molecular weight C resources and are increasingly using anaerobic energy strategies and using less accessible C and P forms. The mixed responses of methanogenesis genes indicate that confounding factors of pore architecture may also be present. Bioindicator analyses, supported many of these findings, affirming their importance in integrative soil monitoring. Future work on this system should involve measurements of methane flux along with metagenomic analyses to determine the identity of the methanogens vegetated and non-vegetated soils. In addition, a successional regrowth of plant matter in the non-vegetated plots may reveal the trajectories by which soil communities can recover from long-term C deprivation.

## Data Availability Statement

The datasets presented in this study can be found in online repositories. The names of the repository/repositories and accession number(s) can be found below: https://www.ebi.ac.uk/ena, PRJEB33898.

## Author Contributions

PG, DF, SC, RG, JM, and DJ conceived and developed the project. PG and DJ led physicochemical analyses, with logistical assistance from DR. JA and SM performed x-ray imagining and analyses. JV generated GeoChip data and was assisted by PG for analyses. PG performed the bioinformatics and statistical analyses with assistance from DF, RG, and JM, and prepared the manuscript. All authors contributed to subsequent revisions and approving the final draft.

## Conflict of Interest

The authors declare that the research was conducted in the absence of any commercial or financial relationships that could be construed as a potential conflict of interest.

## Publisher’s Note

All claims expressed in this article are solely those of the authors and do not necessarily represent those of their affiliated organizations, or those of the publisher, the editors and the reviewers. Any product that may be evaluated in this article, or claim that may be made by its manufacturer, is not guaranteed or endorsed by the publisher.
