# Supplementary material for: Shifts in Soil Structure, Biological, and Functional Diversity Under Long-Term Carbon Deprivation
Source: Front Microbiol. 2021 Sep 14;12:735022. doi: 10.3389/fmicb.2021.735022 (PMC8477002; doi:10.3389/fmicb.2021.735022)
Supplement: Supplementary file 1 [file Data_Sheet_1.DOCX]

**Shifts in soil microbial biological and functional diversity under long-term carbon depletion**

**Supplementary material**

Paul B.L. George*^1,2,3^, David B. Fidler^1^, Joy Van Nostrand^4^, Jonathan A. Atkinson^5^, Sacha J. Mooney^5^, Simon Creer^1^, Robert I. Griffiths^2^, James McDonald^1^, David A. Robinson^2^, Davey L. Jones^1,6^

^1^ *School of Natural Sciences, Bangor University, Deiniol Road, Bangor, Gwynedd, LL57 2UW, UK.*

^2^ *Centre for Ecology & Hydrology, Environment Centre Wales, Deiniol Road, Bangor,*

*Gwynedd, LL57 2UW, UK.*

^3^ *Département de médecine moléculaire, Université Laval, 1050 avenue de la Médecine, Québec, QC, G1V 0A6, Canada*

^4^ *Institute for Environmental Genomics, The University of Oklahoma, Norman, OK 73019, USA.*

*^5^ School of Biosciences, University of Nottingham, Sutton Bonington Campus, LE12 5RD, UK,*

*^6^ SoilsWest, School of Agriculture and Environment, The University of Western Australia, Perth, WA 6009, Australia.*

*Corresponding author. Département de médecine moléculaire, Université Laval, 1050 avenue de la Médecine, Québec, QC, G1V 0A6, Canada. *Email*: [paul.george.1@ulaval.ca](mailto:afp67e@bangor.ac.uk)

**Fig. S1.** Schematic diagram of **a)** non-vegetated and vegetated plot layout as well as **b)** a detailed description of the bare fallow plot design.

** Fig. S2.** Mean pore size distribution collected using X-ray μCT on **a)** whole column data and **b)** aggregate data. Error bars denote standard error of mean.

**Fig. S3.** Conical analyses of principle coordinates ordinations of **A)** prokaryotes; **B)** fungi; and **C)** Protists.

**Fig. S4.** Proportional abundances of trophic modes of fungal OTUs identified by FUNGuild.

**Fig. S5.** Complete differential abundance of prokaryotic indicator taxa of bare fallow and vegetated soils identified using linear discriminant analyses (LDA > 2.5) for 10 year-old soils.

**Fig. S6.** Complete differential abundance of protistan indicator taxa of bare fallow and vegetated soils identified using linear discriminant analyses (LDA > 2.5) for **a)** 1 year-old and **b)** 10 year-old soils.

**Table S1**. Soil porosity from X-ray μCT analysis conducted at whole column and aggregate scale for vegetated vs. 10 year bare fallow only. Mean values (± SE) are presented for both treatments. Significant differences indicated by *** (p<0.001).

|  |  | **Vegetated** | **Non-vegetated** |
| --- | --- | --- | --- |
| **Column scale** | Mean pore size (mm^2^) | 1.47 (± 0.01) *** | 1.30 (± 0.01) |
|  | Porosity (%) | 16.09 (± 3.51) | 6.18 (± 1.55) |
|  | Total Pore Area (mm^2^) | 437745.55 (± 95445.62) | 167986.71 (± 42062.95) |
|  | PSD Ratio (D_10:60_) | 109.76 (± 36.41) | 57.72 (± 12.79) |
|  | Euler number | 27413.0 (± 40231.7) | 88963.0 (± 14565.0) |
| **Aggregate scale** | Mean pore size (mm^2^) | 0.05 (± 0.00) *** | 0.01 (± 0.00) |
|  | Porosity (%) | 10.62 (± 1.14) | 8.58 (± 1.05) |
|  | Total Pore Area (mm^2^) | 206.37 (± 22.24) | 166.90 (± 20.49) |
|  | PSD Ratio (D_10:60_) | 7.17 (± 1.10) | 5.31 (± 0.72) |
|  | Euler number | 25852.5 (± 12911.0) | -9440 (± 12124.6) |
|  |  |  |  |

**Table S2.** Spearmn’s rho values of correlations between richness and soil and environmental variables. *** indicates P < 0.001, ** 0.001 > P < 0.01, * 0.01 > P < 0.05, blank indicates P > 0.05.

| Soil and environmental variables | Taxon | | |
| --- | --- | --- | --- |
|  | Prokaryotes | Fungi | Protists |
| pH | 0.64* | 0.5 | 0.82** |
| Electrical conductivity (μS cm^-1^) | -0.23 | -0.05 | -0.75** |
| Soil CO_2_ flux (μmol m^-2^ s^-1^) | 0.41 | 0.16 | 0.62* |
| Moisture (% dry weight) | 0.22 | 0.3 | -0.42 |
| Total C (%) | 0.71** | 0.8** | 0.4 |
| Total N (%) | 0.62* | 0.52 | 0.03 |
| C: N ratio | 0.24 | 0.5 | 0.6* |
| Nitrate (mg NO_3_^-^ kg^-1^) | 0.4 | 0.29 | -0.24 |
| Ammonium (mg NH_4_^+^ kg^-1^) | 0.32 | -0.12 | -0.19 |
| Available phosphorus (mg P kg^-1^) | 0.2 | 0.13 | 0.27 |
| Calcium (nmol Ca kg^-1^) | 0.65* | 0.32 | 0.11 |
| Potassium (nmol K kg^-1^) | 0.36 | 0.24 | -0.23 |
| Sodium (nmol Na kg^-1^) | 0.17 | 0.3 | 0.68** |
| Magnesium (nmol Mn kg^-1^) | 0.34 | 0.08 | -0.02 |
| Total cations (nmol kg^-1^) | 0.51 | 0.31 | 0.2 |
| Cation exchange capacity (nmol kg^-1^) | 0.34 | 0.1 | -0.02 |
| Temperature (°C) | -0.4 | -0.32 | -0.52 |

Note: Aluminium is not included in these analyses as it could not be detected in the 1-year treatments.

**Table S3.** Spearmn’s rho values of correlations between H’ and soil and environmental variables. *** indicates P < 0.001, ** 0.001 > P < 0.01, * 0.01 > P < 0.05, blank indicates P > 0.05.

| Soil and environmental variables | Taxon | | |
| --- | --- | --- | --- |
|  | Prokaryotes | Fungi | Protists |
| pH | 0.81*** | -0.24 | 0.13 |
| Electrical conductivity (μS cm^-1^) | -0.31 | 0.28 | 0.21 |
| Soil CO_2_ flux (μmol m^-2^ s^-1^) | 0.72** | -0.24 | 0.09 |
| Moisture (% dry weight) | -0.03 | 0.31 | 0.25 |
| Total C (%) | 0.69** | -0.03 | 0.6* |
| Total N (%) | 0.69** | 0.17 | 0.64* |
| C: N ratio | 0.23 | -0.29 | 0.15 |
| Nitrate (mg NO_3_^-^ kg^-1^) | -0.02 | 0.25 | 0.33 |
| Ammonium (mg NH_4_^+^ kg^-1^) | 0.33 | 0.43 | 0.1 |
| Available phosphorus (mg P kg^-1^) | 0.31 | 0.1 | 0.22 |
| Calcium (nmol Ca kg^-1^) | 0.82*** | 0.23 | 0.44 |
| Potassium (nmol K kg^-1^) | 0.18 | 0.21 | 0.49 |
| Sodium (nmol Na kg^-1^) | 0.57* | -0.26 | 0.002 |
| Magnesium (nmol Mn kg^-1^) | 0.26 | 0.26 | 0.29 |
| Total cations (nmol kg^-1^) | 0.83*** | 0.17 | 0.41 |
| Cation exchange capacity (nmol kg^-1^) | 0.26 | 0.27 | 0.33 |
| Temperature (°C) | -0.4 | -0.32 | -0.52 |

Note: Aluminium is not included in these analyses as it could not be detected in the 1-year treatments.

**Table S4.** This Excel sheet contains the output of DESeq2 analyses for prokaryotes.

**Table S5.** This Excel sheet contains the output of DESeq2 analyses for fungi.

**Table S6.** This Excel sheet contains the output of DESeq2 analyses for protists.
